# Effect of Laser Remelting Strategy on the Forming Ability of Cemented Carbide Fabricated by Laser Powder Bed Fusion (L-PBF)

**DOI:** 10.3390/ma15072380

**Published:** 2022-03-23

**Authors:** Decheng Liu, Wen Yue, Jiajie Kang, Chengbiao Wang

**Affiliations:** 1 School of Engineering and Technology, China University of Geosciences (Beijing), Beijing 100084, China; ldccugb@sina.com (D.L.); cbwang@cugb.edu.cn (C.W.); 2 Beijing Geological and Mineral Exploration and Development Group Co., Ltd., Beijing 100050, China; 3 Zhengzhou Institute, China University of Geosciences (Beijing), Zhengzhou 451283, China

**Keywords:** laser powder bed fusion, remelting strategy, surface quality, relative density

## Abstract

Due to the high degree of design freedom and rapid prototyping, laser powder bed fusion (L-PBF) presents a great advantage in the super-hard cemented carbide compared with conventional methods. However, optimizing processing parameters to improve the relative density and surface roughness is still a challenge for cemented carbide fabricated by L-PBF. For this, the effect of the remelting strategy on the forming quality of the L-PBF processed cemented carbide was studied in this article, aiming to explore a suitable process window. The surface quality, relative density, microstructure, and microhardness of the cemented carbide parts fabricated under a single melting and remelting strategy were compared. The results showed that the remelting strategy could efficiently improve the specimens’ surface quality and relative density. Besides, the cracks were not obviously aggravated, and the WC grains could distribute more homogeneously on the binder matrix under the remelting strategy. Therefore, the microhardness showed an improvement compared to the single melting strategy.

## 1. Introduction

Laser powder bed fusion (L-PBF), as an extremely promising embranchment of additive manufacturing (AM) techniques, utilizes a powder bed system to supply the metallic powder and laser beam to selectively melt metallic powder in a layer-wise mode, so it can rapidly build complex-shaped metallic parts according to the CAD model [1,2]. The high amount of design freedom and manufacturing flexibility of L-PBF present the advantage of low cost and high efficiency over traditional methods in manufacturing non-standard and personalized parts. To date, L-PBF has been applied in multiple industries, including aerospace, biomedical, tooling, and automotive industries [3,4]. Meanwhile, dozens of materials have been successfully fabricated by L-PBF [5].

Although L-PBF technology presents significant advantages in fabricating complex geometric parts compared to conventional technologies, it still has many drawbacks that need to be overcome, including the low surface quality and pore formation [6,7]. Previous studies revealed that processing parameters such as laser power, scanning speed, and scanning strategy directly affect these qualities of the L-PBF. Optimizing these process parameters to obtain parts that satisfy the engineering application is critical to L-PBF processing [8,9]. In the work of Read et al. [10], the effect of scanning speed and laser power on the densification of an L-PBF AlSi10Mg alloy was studied. The work revealed that decreasing the laser power or increasing the scanning speed would reduce the laser energy input on the metal powders, thus leading to an incomplete melt of materials and the formation of pores on the parts. Similar phenomena were also demonstrated by Koutiri et al. [11] and Moussaoui et al. [12]. In addition, the insufficient energy input caused by a high scanning speed and low lase power resulted in irregular single tracks with various defects such as balling and discontinuity, which tended to be distorted and loosen the bonding between adjacent laser tracks, thus producing a coarser surface [13,14,15]. The remelting strategy could also significantly influence the pore formation and surface morphology. Liu et al. [16] adopted a laser remelting strategy in the L-PBF fabrication of AlSi10Mg, and it revealed that the laser remelting strategy extends the building time, which was conducive to good metallurgical bonding between the molten pools, thus improving the surface quality and relative density. Yu et al. [17] found that the additional remelting of processed layers helped to obtain the highest relative density and lowest surface roughness for the L-PBF-W materials. Yasa et al. [18] and Qiu et al. [19] proved that the bonding between the layers could be significantly improved by the remelting strategy, thus decreasing the porosity in the specimens. Wei et al. [20] found that the remelting strategy could improve the surface quality and density and enhance the mechanical properties, including the microhardness and tensile strength.

Due to their excellent wear-resistance and ultra-high hardness, cemented carbide materials have covered a wide range of applications in many industries where they face extremely harsh working conditions, such as geological drilling, machining, tunneling, and molding [21,22]. However, the traditional sintering method can only fabricate the cemented carbide parts with symmetrical and simple geometry. Besides, the ultra-high hardness makes the post-machining of cemented carbide parts costly and time-consuming [23]. Therefore, to achieve rapid fabrication of the cemented carbide parts with a complex shape to satisfy the requirements of the advanced industry, researchers are attempting to employ L-PBF technology for manufacturing cemented carbide parts [24,25]. However, the existing research found many severe defects during the L-PBF processing of cemented carbide, such as the low relative density, cracks, and high surface roughness [26,27]. On the one hand, these defects were primarily induced by the low flowability and brittleness of materials [28,29]. Khmyrov et al. [30] proved that brittle-phase W_3_Co_3_C was formed during the L-PBF process, which was responsible for cracking. On the other hand, studies on the processing optimization of L-PBF cemented carbide are fewer than that of other materials. Gu et al. [31] fabricated the cemented carbide by L-PBF with different scanning speeds. Domashenkov et al. [32] studied the effect of the laser power and scanning speed on the microstructural evolution of L-PBF cemented carbide. Uhlmann et al. [23] and Chen et al. [25] changed the hatching space in the L-PBF fabrication of cemented carbide. However, more kinds of processing parameters should be considered, particularly the remelting scanning strategy, which is highly effective to improve the forming quality of parts fabricated by L-PBF.

Therefore, in this study, the remelting strategy was applied in the L-PBF processing of cemented carbide, aiming to improve the parts’ relative density and surface roughness. To find the suitable process parameters to match the remelting strategy, the effect of different laser powers and scanning speeds on the forming quality of cemented carbide were studied.

## 2. Experimental Details

### 2.1. Powder Characteristics

**Figure 1:**
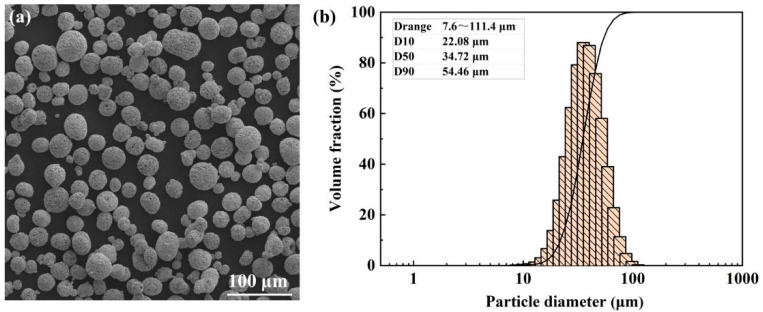
The WC-17Co powders: (**a**) The morphology, (**b**) the particle size distribution.

This study employed commercial spherical WC-17Co (WC: 83, Co: 17) powders supplied from the Shenyang Institute of Nonferrous Metals. The morphology of the powders was characterized by the Phenom Prox scanning electron microscope (SEM, Eindhoven, The Netherlands), and the particle size distribution of powders was measured by the laser particle size analyzer (Bettersize 3000 Plus, Dandong, China). As shown in Figure 1, the WC-17Co powders present a spherical shape with a D50 value of 34.72 μm. In addition, the flowability of the WC-17Co powders was determined using the static Angle of Repose (AOR), as shown in Table 1. The AOR of the WC-17Co powders, determined based on a flow tester powder comprehensive characteristic tester (Bettersize 100, Dandong, China), was 26.3°, proving good flowability for the powders.

### 2.2. L-PBF Process

All specimens were fabricated by the L-PBF system (EOSINT M280, Munich, Germany), which has a fiber laser with a maximum power of 400 W and a spot size of 100 μm. A pure nickel substrate was used in this fabrication because our previous study had proven that the nickel substrate presented excellent adhesive bonding to the WC-17Co materials for a wide range of laser energy inputs [27]. Moreover, the substrate was pre-heated to 80 °C before the fabrication. The manufacturing process was controlled by the PSW software and operated in an Argon (Ar) protection environment, which was used to ensure an oxygen content of less than 0.1% during the process.

**Figure 2:**
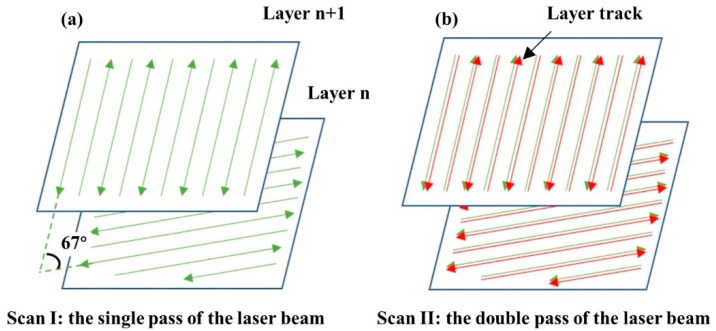
The scanning strategy of the specimens: (**a**) Single melting strategy, (**b**) remelting strategy.

A single melting strategy (Figure 2a) and a remelting strategy (Figure 2b), with a rotation angle of 67° between layers, were used. The processing parameters of the specimens are shown in Table 2.

### 2.3. Characterization

The densities of the specimens for densification characterization were obtained by the Archimedes principle for three measurements and are presented as a relative density to the WC-17Co (13.85 g/mm^3^). The surface morphologies of the specimens were characterized by SEM. The surface roughness (Ra) was measured by the laser scanning confocal microscope (Olympus 4000, Tokyo, Japan) from the three-dimensional morphologies of specimens according to the ISO 25178 standard. The phase composition of the polished surface of the specimens was determined employing X-ray diffraction (XRD, Smartlab, Tokyo, Japan). The Vickers hardness of the samples was determined using a hardness tester (VTD512, Shenzhen, China) at a load of 500 gf applied for 15 s. An average of at least 10 readings was reported.

## 3. Results

### 3.1. Surface Morphology

**Figure 3:**
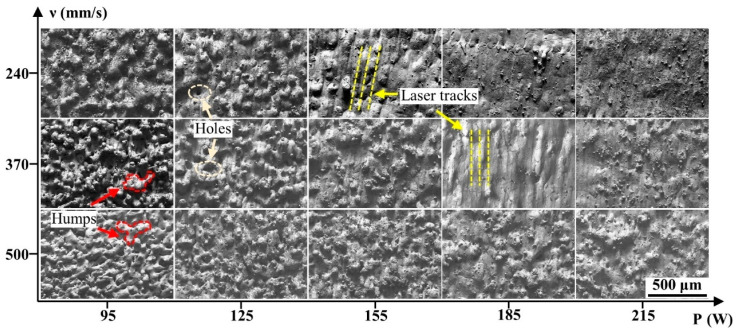
The top surface morphology of the cemented carbide parts formed under the Scan I strategy.

Figure 3 shows the surface morphologies of the blocks formed at different processing parameters in the Scan I strategy. It revealed that the surface morphologies were highly dependent on the laser powers and scanning speeds. At the scanning speed of 500 mm/s, the blocks had considerably coarse morphologies, which were occupied by severe humps and larger quantities of holes. Besides, no regular scanning tracks were found on these surfaces because the serious defects disrupted the formation of the regular scanning tracks. It could also be found that increasing the laser power could improve the surface quality; the surface morphology of the block formed at 215 W was better than that of the block formed at 95 W, although this surface was still rough. When the scanning speed decreased to 370 mm/s, the surface quality had an effective improvement. When the laser power was 185 and 215 W, the surface consisted of regular scanning tracks that had less severe humps and holes, thus forming a relatively flat surface compared to the surface at a scanning speed of 500 mm/s. As the scanning speed continued to decrease to 240 mm/s, the surface quality was still improved. However, the surface quality was not effectively improved at laser powers of 185 and 215 W compared to that at a scanning speed of 370 mm/s, meaning that the continuous increase in laser power or decrease in scanning speed could no longer optimize the surface quality.

**Figure 4:**
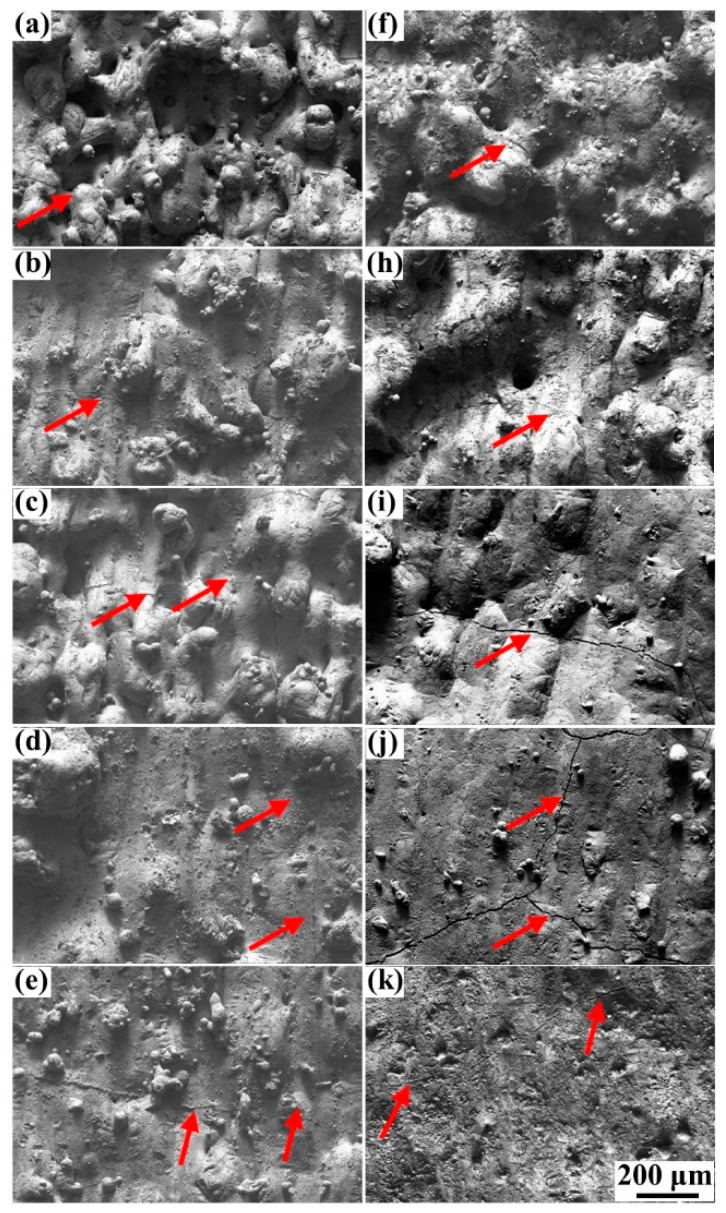
The high-magnification SEM pictures of the top surface morphology corresponding to Figure 3: (**a**–**e**) Scanning speed of 370 mm/s, laser power from 95 to 215 W; (**f**–**k**) scanning speed of 240 mm/s, laser power from 95 to 215 W.

The high-magnification SEM pictures of the surface morphology at 240 mm/s and 370 mm/s are shown in Figure 4. These high-magnification pictures helped to find the tiny cracks on the blocks. It revealed that the cracks were inevitably generated on all specimens, similar to the previous studies [23,27]. The formation of cracks was mainly attributed to the brittleness of the cemented carbide. In addition, the cracks on the blocks formed at the low scanning speed of 240 mm/s were more serious compared to that of the blocks formed at 370 mm/s, particularly at a laser power from 185 to 215 W (Figure 4i–k). These cracks were wider and more continuous, in accordance with Equation (1):

(1) $$D=\frac{P}{\nu ht}$$

where D is the laser energy density, P is the laser power, ν is the scanning speed, h is the hatching space, and t is the layer thickness [33]. The low scanning speed and high laser power produced a high laser energy input. Therefore, the result demonstrated that the overly high laser energy input not only had little effect on improving the surface quality but would also cause more severe cracks.

**Figure 5:**
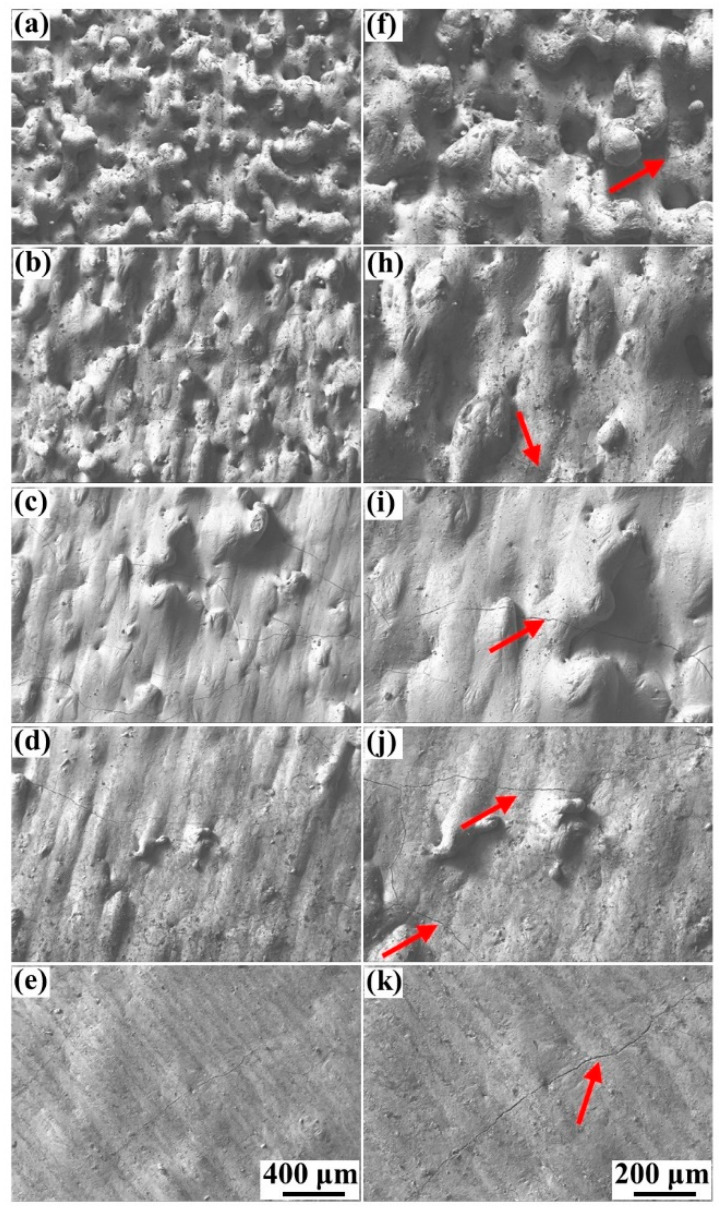
The top surface morphology of the specimens formed at 370 mm/s under the Scan II strategy: (**a**–**e**) Laser power from 95 to 215 W, (**f**–**k**) the large magnification corresponding to (**a**–**e**).

The surface morphology of the samples formed at the scanning speed of 370 mm/s and the Scan II strategy are shown in Figure 5. It exhibited that the defects of humps, holes, and irregular scanning tracks on the surface decreased sharply compared to that of the specimens formed at both 370 mm/s and 240 mm/s without the remelting strategy. The surface morphology approached a common smooth plane especially when the specimens were formed at 185 and 215 W under the Scan II strategy, meaning the remelting strategy could effectively improve the surface quality. According to Yu et al. [17], adopting laser source remelting on the deposited layers meant applying an additional laser energy input, equal to the original laser energy input, on the specimens, meaning the remelting strategy was another method to improve the laser energy input. However, Figure 5c–e showed that the cracks on the surface of these specimens were merely slightly aggravated in comparison with the samples fabricated at 370 mm/s under the Scan I strategy (Figure 4c–e). However, it was still much better than the samples formed under the high laser energy density (Figure 4i–k). The result demonstrated that the remelting strategy might provide a more suitable process window than increasing the laser energy density.

**Figure 6:**
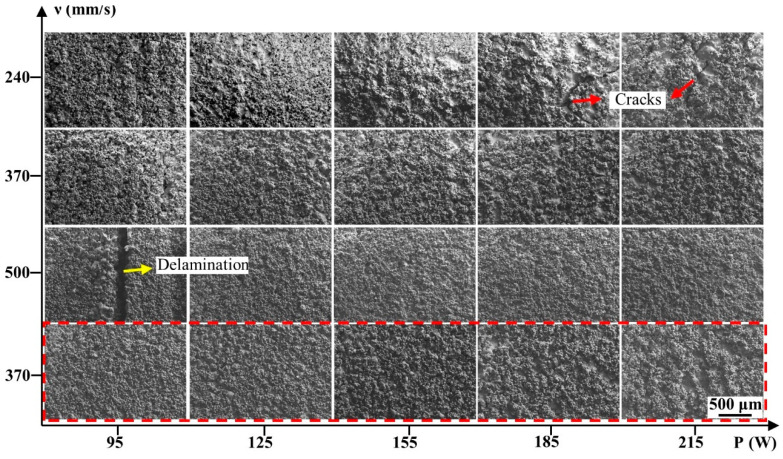
The front surface morphology of the specimens formed at different process parameters: The samples in the red dashed box were formed under the Scan II strategy.

The front surface morphology of the specimens was also observed by SEM, as shown in Figure 6. It revealed that a large quantity of the powders adhered to all of the blocks’ surfaces. This phenomenon, similar to the previous studies [34], was inevitable because the liquid phase would sinter adjacent powders to form the molten pools. Although the existence of these powders led to difficulty in observing the actual surface morphology of these blocks, it was still found that apparent delamination appeared at the surface when the samples formed at 95 W, 500 mm/s, and severe cracks on the surface when the samples formed at 185–215 W, 240 mm/s. In contrast, other blocks had an intact surface without apparent defects.

### 3.2. Surface Roughness

**Figure 7:**
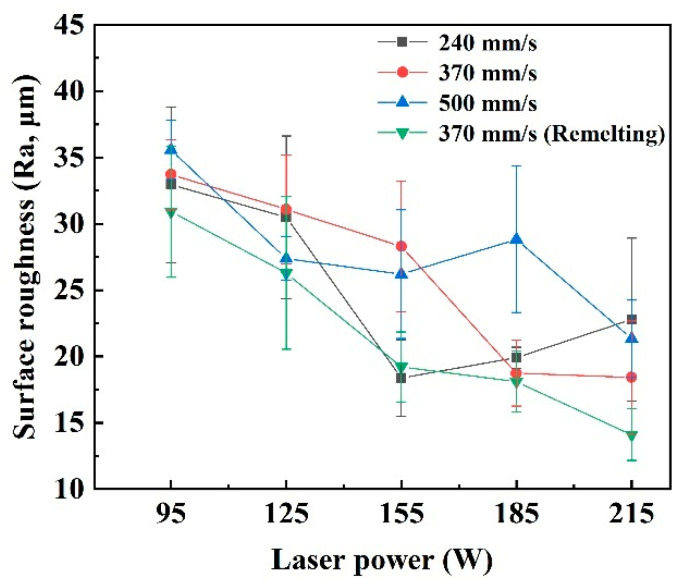
The surface roughness of the blocks formed under different process parameters.

The surface roughness of the blocks formed under different process parameters is shown in Figure 7, where the standard deviation is plotted as error bars. It directly proved that the remelting strategy provided a great improvement in the surface quality of the cemented carbide parts. When under the Scan I strategy, the surface roughness of the blocks presented a decreasing tendency with the increase in laser power or the decrease in scanning speed. With the scanning speed decreasing from 500 to 370 mm/s and laser power increasing from 95 to 215 W, the surface roughness decreased from 35.57 to 18.43 μm. When the scanning speed decreased to 240 mm/s, the best surface roughness was still obtained at the high laser power of 185 and 215 W, but the Ra value was not efficiently decreased compared to that of the blocks formed at 370 mm/s. Meanwhile, the surface roughness of the blocks was significantly decreased when the Scan II strategy was applied at 370 mm/s. Moreover, the best surface roughness of 14.10 μm was obtained. In addition, it could also be found that the small error bars were obtained when the surface roughness was good due to low fluctuation of the average roughness value, also proving the excellent surface quality.

**Figure 8:**
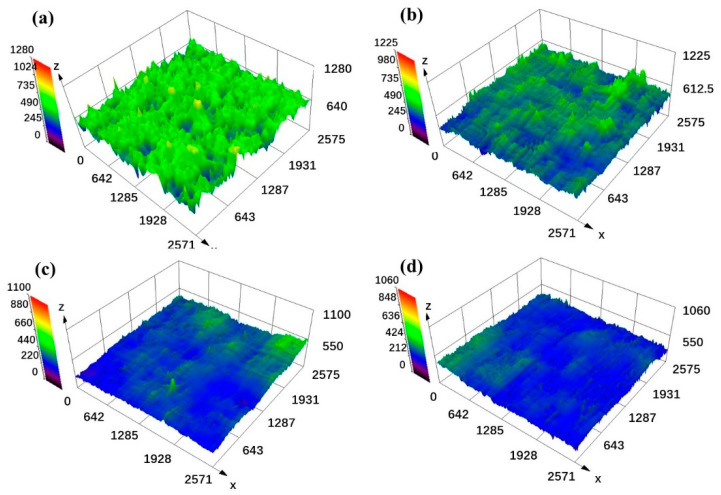
The three-dimensional surface morphology of the cemented carbide blocks formed under different process parameters, (**a**) 95 W, 500 mm/s, scan I; (**b**) 125 W, 370 mm/s, scan I; (**c**) 215 W, 370 mm/s scan I; (**d**) 215 W, 370 mm/s, scan II.

**Figure 9:**
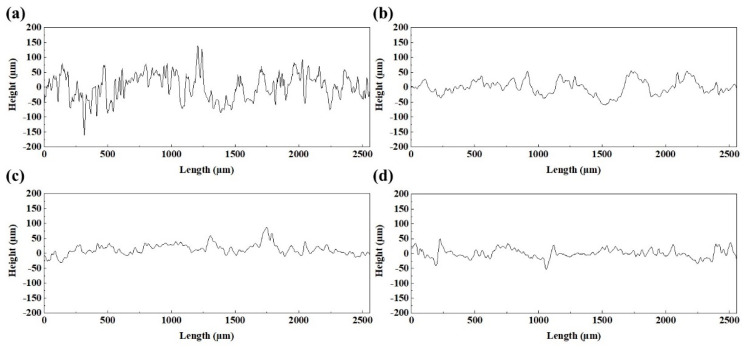
The surface profiles were obtained along the x-direction on the surface in Figure 8: (**a**) 95 W, 500 mm/s, scan I; (**b**) 125 W, 370 mm/s, scan I; (**c**) 215 W, 370 mm/s scan I; (**d**) 215 W, 370 mm/s, scan II.

Figure 8 shows the three-dimensional surface morphology of the blocks under different process parameters, and the corresponding surface profile of these blocks is shown in Figure 9. The results revealed that the rugged surface formed under low laser energy input (Figure 8a,b) induced a highly fluctuating surface profile. According to the roughness calculation formula (2)

(2) $$Ra=\frac{1}{L}\int_{0}^{L}\left|f(x)\right|dx$$

L is the measured length and f(x) is the surface height from the average height of the surface (0 μm in Figure 9) [35]. Therefore, the strong fluctuant surface profile with a large height led to the high surface roughness, while the smooth surface profile with a small height (Figure 9c,d) corresponds to the low surface roughness. As shown in Figure 3, defects such as humps and holes that formed on the blocks directly caused this coarse surface morphology, which was not conducive to achieving low surface roughness.

### 3.3. Densification Behaviors

**Figure 10:**
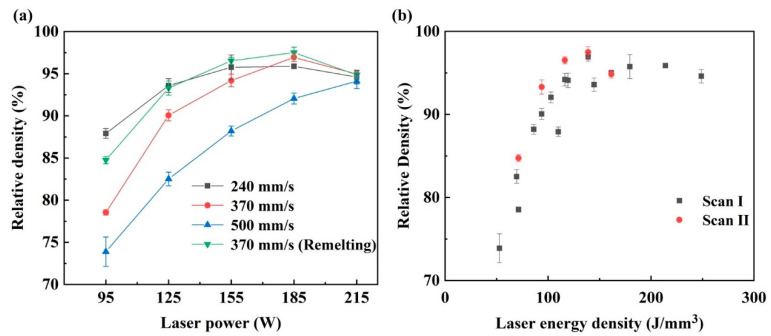
The relative densities of the cemented carbide parts: (**a**) The relative density at different process parameters; (**b**) the relative density at different laser energy densities.

The relative density of the samples formed under different laser processing parameters and laser energy densities are shown in Figure 10. From Figure 10a, it was found that the relative density of the sample was closely related to the process parameters. At the scanning speed of 500 mm/s, the relative density increased from 74.89% to 94.11%, with the laser power increasing from 95 W to 215 W. When the scanning speed decreased to 370 mm/s, the relative density showed an obvious increment. The relative density still presented an increasing trend when the laser power increased from 95 W to 185 W, corresponding to the relative density increase from 78.55% to 96.44%. However, it decreased to 93.64% at the laser power of 215 W. Instead, the relative density did not increase when the scanning speed decreased to 240 mm/s, particularly at the high laser powers of 185 W and 215 W, whereby the relative density was 95.87% and 94.61%, lower than that at the scanning speed of 370 mm/s. The variation of the laser energy density caused this phenomenon. As shown in Figure 10b, a poor relative density was achieved at the low energy density, and increasing the laser energy density improved the relative density. However, the overly high laser energy density worsened the densification behavior. For comparison, it was found that the remelting strategy applied at the scanning speed of 370 mm/s could present better densification behavior for the specimens. Under the Scan II strategy, the relative density showed a similar increasing tendency from 95 to 185 W to the Scan I strategy, and the values were higher than that of Scan I, and the best relative density of 97.50% was achieved at 185 W. Besides, there is also a decrease in the laser power of 215 W, similar to the samples formed under the Scan I strategy.

**Figure 11:**
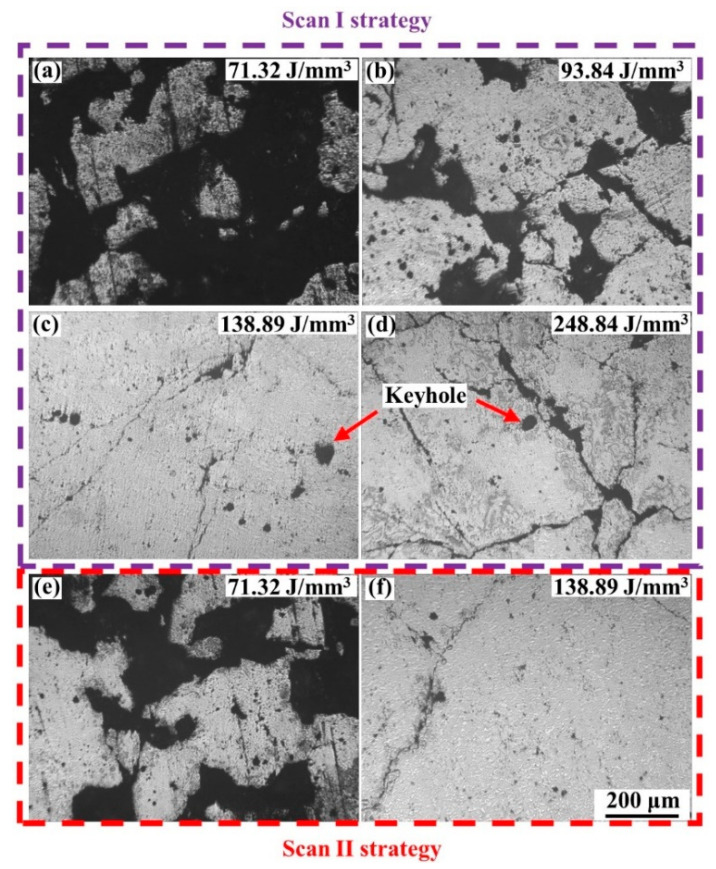
The polished surface of the cemented carbide blocks formed under different laser energy densities: (**a**) 71.32 J/mm^3^ Scan I; (**b**) 93.84 J/mm^3^ Scan I; (**c**) 138.89 J/mm^3^ Scan I; (**d**) 248.84 J/mm^3^ Scan I; (**e**) 71.32 J/mm^3^ Scan II; (**f**) 138.89 J/mm^3^ Scan II.

Figure 11 shows the polished surface of the blocks, and it exhibited that the pores and cracks on blocks induced the low relative density. When the laser energy density was low, large amounts of irregular pores formed on the blocks, leading to a low density. With the increase in the laser energy density, the number and size of irregular pores decreased, improving the relative density. However, some almost circular pores with a size of approximately 30–50 μm appeared, as shown in Figure 11c,d, when the laser energy density was high. These circular pores were keyholes, which were caused by the collapse of the vapor cavity that formed by the evaporation of the materials under a high laser energy density [36]. In addition, the cracks became more evident, and as shown in Figure 11d, the cracks were long and wide. Both the keyholes and serious cracks could also induce the low density of the blocks. Besides, the remelting strategy was able to effectively restrain the formation of pores at a low energy density, and the cracks merely became slightly worse compared to that under the single melting strategy. Therefore, a better relative density was achieved.

Low energy density would lead to insufficient liquid materials in the molten pools. Besides, according to Equation (3),

(3) $$\eta_{\nu }=\frac{16}{15}\sqrt{\frac{m}{kT}}\sigma$$

where $\eta_{\nu }$ is the dynamic viscosity of the liquid phase in the molten pool, m is the atomic mass, k is the Boltzmann constant, T is the molten pool temperature, and σ is the surface tension, which is inversely proportional to T [37], the low temperature of the molten pools caused by the low laser energy density would induce high viscosity of the liquid materials. Therefore, the wettability and spreadability of the liquid phase worsened. The metal liquid would not well spread and wet the metal powders, which limited the formation of regular and continuous scanning tracks, as shown in Figure 3, and the scanning tracks were irregular and discontinuous. Once these irregular scanning tracks form, the overlap between the adjacent scanning tracks would be loose and irregular. The irregular pores would form under these conditions. With the laser energy density increase, the molten pools would have more liquid materials with good wettability, forming regular and continuous laser tracks. The overlap between the adjacent scanning tracks was tight and regular. The formation of pores would be restrained. However, the thermal stress would be increased when the laser energy density increased, which aggravated the crack defects [23]. When the blocks were formed under the Scan II strategy, the remelting of the molten pools lengthened the solidification time of liquid materials [16]. The liquid phase had more time to spread and wet more materials, which contributed to regular scanning tracks with a large width, thereby achieving the tight overlap between adjacent laser tracks. The formation of pores would be efficiently restrained. Moreover, compared with the increment in the laser energy density by decreasing the scanning speed to 240 mm/s under the Scan I strategy, the remelting strategy had lower energy density input, which would decrease the thermal stress in the L-PBF processing and restrain the formation of serious cracks.

### 3.4. Microstructures

**Figure 12:**
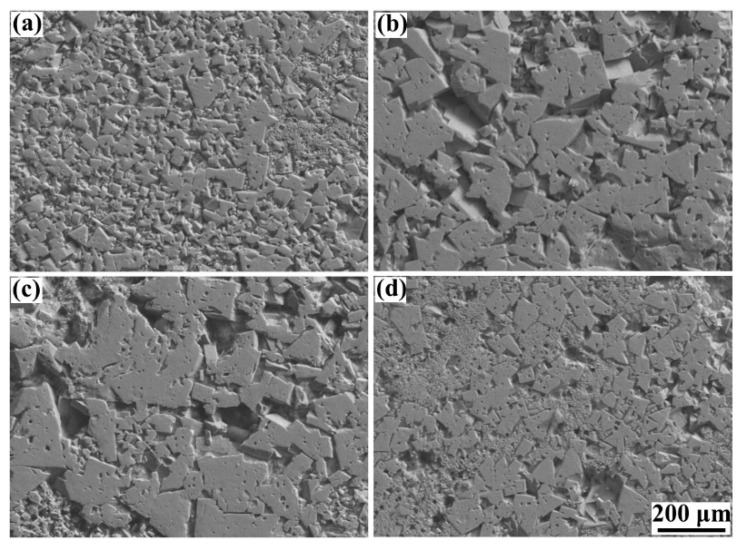
The microstructure of cemented carbide blocks formed under different laser energy densities and scanning strategies: (**a**) Scan I, 116.37 J/mm^3^; (**b**) Scan I, 161.41 J/mm^3^; (**c**) Scan I, 248.84 J/mm^3^; (**d**) Scan II, 116.37 J/mm^3^.

**Figure 13:**
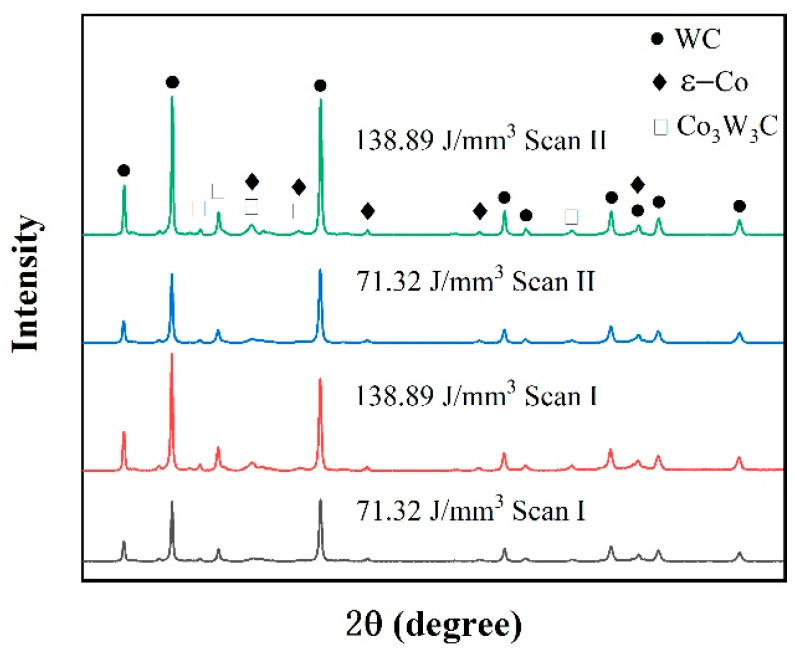
XRD spectrum of specimens formed under different process parameters and scanning strategies.

The microstructures of the blocks formed under different laser energy densities are shown in Figure 12, and it shows that the polygon-structured WC grains are distributed on the Co binder matrix, and characteristics such as the grain size and homogeneity of WC grains are highly dependent on the laser energy density. At the low energy density of 116.37 J/mm^3^, many of the tiny WC grains clustered on the Co binder matrix presented an inhomogeneous distribution. This result was caused by the low temperature of the liquid phase. According to Pasquet et al. [38], low laser energy density might produce a low temperature of molten pools that only exceeds the melting point of Co binder materials (1457 °C) but is lower than the melting point of WC (2870 °C), which was not conducive to grain nucleation. Thus, the microstructure presented inferior characteristics. When the laser energy density increased to 161.41 J/mm^3^, sufficient energy density could produce a sufficiently high temperature for the sintering and melting of all the materials. Therefore, it was found that the WC grains with large sizes were distributed uniformly on the binder matrix. Due to the overly high temperature of the liquid phase, the grain size continued to increase at the energy density of 248.84 J/mm^3^, and an apparent coarsening characteristic of WC grains was observed, which, in turn, was considered harmful for the performance of the cemented carbide parts [25]. Besides, it was found that both the grain size and distribution of WC grains could be improved by applying the remelting strategy by comparing the results of Figure 10a,d. The remelting strategy allowed for a long duration for the transformation from the liquid phase to the solid phase, which was beneficial to grain nucleation. The XRD result of the phase composition of the blocks, shown in Figure 13, also proved these phenomena. The results showed that, in addition to the WC phase and the hexagonal ε-Co phase, there was a Co_3_W_3_C phase in the specimens, which was similar to the results of Khmyrov et al. [30]. Furthermore, the Co_3_W_3_C phase would promote cracking. Moreover, it was found that the peak intensity of all phases increased when a high laser energy density was applied or the remelting strategy was adopted, leading to good grain nucleation, which was caused by the high temperature and long solidification time.

### 3.5. Microhardness

**Figure 14:**
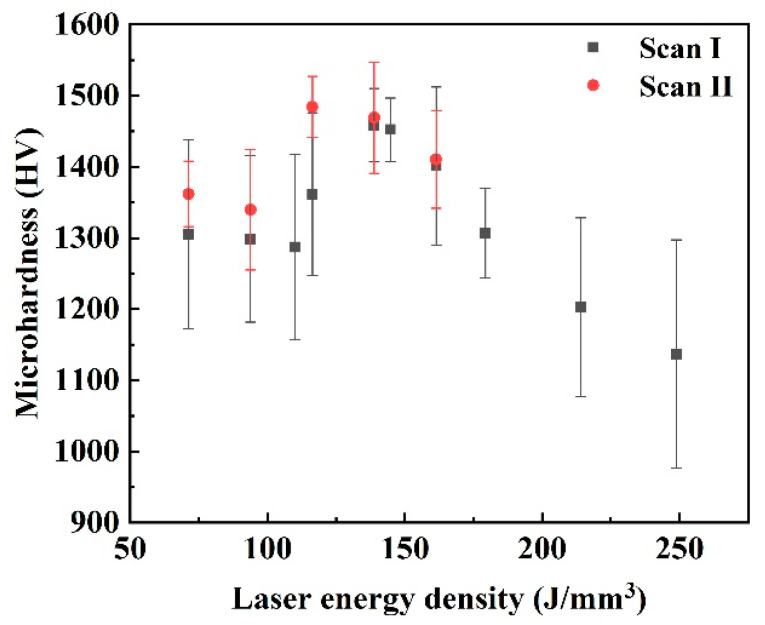
The microhardness of the cemented carbide blocks formed under different laser energy densities.

Figure 14 shows the microhardness of the blocks formed under different laser energy densities and scanning strategies. Under both the Scan I strategy and Scan II strategy, the microhardness presented a variable tendency that increased first and then decreased. Under the Scan I strategy, the lowest microhardness was around 1270–1300 HV when the laser energy density was below 109 J/mm^3^. The high microhardness was around 1401.2–1458.2 HV when the laser energy density was 138.89 to 161.41 J/mm^3^. When the laser energy density continued to increase, the microhardness decreased sharply. The lowest value of 1136.8 HV was obtained at the laser energy density of 248.84 J/mm^3^. Under the Scan II strategy, the high microhardness was around 1410.4–1484.4 HV at the laser energy density of 116.37–161.41 J/mm^3^. This phenomenon closely depended on the relative density and microstructure of the cemented carbide blocks. The high relative density with the WC grains uniformly distributed on the Co binder matrix contributed to a higher microhardness.

## 4. Conclusions

This study applied the remelting strategy in the L-PBF processing of cemented carbide parts, aiming to improve the formation quality. By combining different laser energy densities and the remelting strategy, the specimens’ density, surface quality, microstructure, and microhardness were characterized to find the optimal process window. The general conclusions can be summarized as follows:

1. Irregular scanning tracks formed under a low laser energy density constituted a coarse surface. Increasing the laser energy density and using the remelting strategy could decrease the defects of scanning tracks, thus improving the surface quality. However, the overly high laser energy input more easily aggravated the formation of cracks.
2. The surface roughness of the cemented carbide specimens widely varied from 35.57 to 18.38 μm at the single melting strategy. In contrast, the remelting strategy improved the best surface roughness to 14.10 μm.
3. Under the single melting strategy, the large number of pores induced by the low laser energy density and the serious cracks induced by the overly high laser energy density led to a poor relative density for the specimens. However, under the remelting strategy, the pores were decreased effectively on the specimens at a low energy density. The cracks on the blocks did not worsen, in contrast to the single melting strategy, thus obtaining the best relative density of 97.50%.
4. The size and homogeneity of WC grains could be effectively improved by increasing the laser energy density or using the remelting strategy. However, an overly high laser energy density would lead to coarse grains.
5. The best microhardness of the specimens formed at the single melting strategy was approximately 1401.2–1458.2 HV. In contrast, the best microhardness of approximately 1410.4–1484.4 HV was obtained with the remelting strategy.
6. When the cemented carbide specimens formed under a laser energy density from 116.37 to 161.41 J/mm^3^ with the remelting strategy, good formation quality of the specimens could be obtained.

## Figures and Tables

**Table 1:** Classification of flowability by the AOR.

AOR	Flowability
20° < α < 30°	Very free-flowing
30° < α < 38°	Free-flowing
38° < α < 45°	Fair to passable flow
45° < α < 55°	Cohesive
55° < α < 70°	Very cohesive

**Table 2:** L-PBF processing parameters of the cemented carbide specimens.

Sample	Laser Power (W)	Scanning Speed (mm/s)	Hatching Space (μm)	Layer Thickness (μm)	Scanning Strategy
I	95, 125, 155, 185, 215	240, 370, 500	90	40	Scan I
II	95, 125, 155, 185, 215	370	90	40	Scan II

## Data Availability

The raw data required to reproduce these findings cannot be shared at this time as the data also form part of an ongoing study.
